# Profiling placental and fetal DNA methylation in human neural tube defects

**DOI:** 10.1186/s13072-016-0054-8

**Published:** 2016-02-16

**Authors:** E. Magda Price, Maria S. Peñaherrera, Elodie Portales-Casamar, Paul Pavlidis, Margot I. Van Allen, Deborah E. McFadden, Wendy P. Robinson

**Affiliations:** Child and Family Research Institute, 950 W 28th Ave, Vancouver, BC V5Z 4H4 UK; Dept of Medical Genetics, University of British Columbia, C201-4500 Oak St, Vancouver, BC V6H 3N1 UK; Dept of Obstetrics and Gynaecology, University of British Columbia, C420-4500 Oak St, Vancouver, BC V6H 3N1 UK; Centre for High-Throughput Biology, University of British Columbia, 2185 East Mall, Vancouver, V6T 1Z4 UK; Dept of Psychiatry, University of British Columbia, 2255 Wesbrook Mall, Vancouver, BC V6T 2A1 UK; Dept of Pathology and Laboratory Medicine, Rm G227-2211, Wesbrook Mall, Vancouver, BC V6T 2B5 UK

**Keywords:** Neural tube defects (NTDs), Spina bifida, Anencephaly, Illumina HumanMethylation450 BeadChip, 450k array, Epigenome-wide association study (EWAS), DNA methylation (DNAm)

## Abstract

**Background:**

The incidence of neural tube defects (NTDs) declined by about 40 % in Canada with the introduction of a national folic acid (FA) fortification program. Despite the fact that few Canadians currently exhibit folate deficiency, NTDs are still the second most common congenital abnormality. FA fortification may have aided in reducing the incidence of NTDs by overcoming abnormal one carbon metabolism cycling, the process which provides one carbon units for methylation of DNA. We considered that NTDs persisting in a folate-replete population may also occur in the context of FA-independent compromised one carbon metabolism, and that this might manifest as abnormal DNA methylation (DNAm). Second trimester human placental chorionic villi, kidney, spinal cord, brain, and muscle were collected from 19 control, 22 spina bifida, and 15 anencephalic fetuses in British Columbia, Canada. DNA was extracted, assessed for methylenetetrahydrofolate reductase (*MTHFR*) genotype and for genome-wide DNAm using repetitive elements, in addition to the Illumina Infinium HumanMethylation450 (450k) array.

**Results:**

No difference in repetitive element DNAm was noted between NTD status groups. Using a false discovery rate <0.05 and average group difference in DNAm ≥0.05, differentially methylated array sites were identified only in (1) the comparison of anencephaly to controls in chorionic villi (*n* = 4 sites) and (2) the comparison of spina bifida to controls in kidney (*n* = 3342 sites).

**Conclusions:**

We suggest that the distinctive DNAm of spina bifida kidneys may be consequent to the neural tube defect or reflective of a common etiology for abnormal neural tube and renal development. Though there were some small shifts in DNAm in the other tested tissues, our data do not support the long-standing hypothesis of generalized altered genome-wide DNAm in NTDs. This finding may be related to the fact that most Canadians are not folate deficient, but it importantly opens the field to the investigation of other epigenetic and non-epigenetic mechanisms in the etiology of NTDs.

**Electronic supplementary material:**

The online version of this article (doi:10.1186/s13072-016-0054-8) contains supplementary material, which is available to authorized users.

## Background

Neural tube defects (NTDs) are a spectrum of severe and often lethal congenital abnormalities that arise in the first month of pregnancy from a failure of the flat neural plate to elevate, fold, and close in the developing embryo [1, 2]. Folic acid (FA, synthetic folate) fortification and supplementation programs in more than 80 countries around the world [3] were initiated in response to mounting evidence that the incidence of NTDs could be lowered by increasing maternal pre- and perinatal FA intake [4]. Despite a 40 % decline in the incidence of NTDs [5], a 2007 Canadian post FA fortification survey reported that the most common NTDs—spina bifida (SB, caudal failure of neural tube closure) and anencephaly (AN, cranial failure of neural tube closure)—were still prevalent at respective rates of 0.41 and 0.36 per 1000 live-and stillbirths [6]. NTDs remain one of Canada’s most common congenital abnormalities [5].

The mechanism through which FA helps to prevent NTDs is unclear. Folate is a substrate for the activation of methyl groups in one-carbon metabolism (OCM), a biochemical pathway comprising two intersecting cycles: the DNA cycle (producing nucleotide precursors) and the methylation cycle (producing methyl donors) [7]. A hypothesis of altered capacity for DNA methylation (DNAm) has been proposed as the mechanism underlying FA prevention of NTDs [8]. The basis of this hypothesis was the observation of increased incidence of NTDs in association with maternal and fetal SNPs in methylenetetrahydrofolate reductase (*MTHFR*) [9], the enzyme at the intersection of the DNA and methylation cycles. These *MTHFR* variants result in reduced enzymatic activity [10, 11] which is expected to shift OCM toward the DNA cycle, restricting methylation capacity and leading to abnormal patterns of DNAm [8, 12]. Studies in mice demonstrate that the de novo DNA methyltransferase, Dnmt3b, is expressed in the elevating neural folds during neural tube formation, and that Dnmt3b null mutants develop NTDs [13]. Research from countries without FA fortification also lends support to the methylation hypothesis. A slight reduction in genome-wide DNAm was noted in NTD cases in China [14–16], in addition to changes in NTD DNAm at specific genes relevant to fetal development in China, Belgium, and the Netherlands: imprinted [17], planar-cell polarity [18, 19], HOX [20], and folate receptor genes [21]. Some of the findings are, however, undermined by small magnitude of change in DNAm, a lack of statistical correction for multiple comparisons, and use of peripheral tissues. Despite research and policy changes, NTDs remain the second most common congenital abnormality in many parts of the world [22], and the etiology of persisting cases is unknown.

In this study, we profiled DNAm of NTDs in British Columbia (B.C.), Canada, a population in which folate deficiency is rarely observed [23]. However, vitamin B_12_ insufficiency (a cofactor in OCM) has been noted in Canadian women of childbearing age [24] and low maternal serum B_12_ levels have been associated with an increased risk for NTDs [25, 26]. We therefore considered that in folate replete populations, the same pathways through which FA fortification has contributed to reducing the incidence of NTDs may be dysregulated, but by a different mechanism, and also manifest as abnormal NTD DNAm. A unique set of multiple tissues was collected from second trimester (14–26 weeks) human fetuses with spina bifida (SB, *n* = 22) or anencephaly (AN, *n* = 15) in addition to controls (CON, *n* = 19), to explore DNAm using repetitive elements and the Illumina Infinium HumanMethylation450 BeadChip (450k array) platform. The 450k array assesses DNAm at >450,000 CpG sites throughout the genome, with coverage of 99 % of RefSeq genes and 96 % of CpG islands, allowing the user to obtain a highly accurate snapshot of DNAm at ~1.7 % of all CpGs [27]. We reasoned that observed changes in DNAm in NTDs might be restricted to a single disease-affected tissue (spinal cord or brain) or support tissue (chorionic villi). Alternatively, abnormal DNAm may be present in multiple tissues (including those peripheral to the defect, like kidney or muscle), as an imprint of an insult early in embryo development. Due to the essential role that products of OCM play in cell proliferation, differentiation, and migration [12, 28], NTDs might be associated with (1) changes in DNAm at a specific subset of loci (which may contribute to the development of NTDs or be a direct consequence of a causative pathway) or (2) widespread and/or non-specific changes in DNAm.

## Methods

### Ethics approval

Ethics approval for this study was obtained from the University of British Columbia Children’s and Women’s Research Ethics Board (certificate number: H10—1028). Written consent was obtained for cases ascertained prior to pregnancy termination (*n* = 8). For cases obtained retrospectively from pathological autopsy specimens (*n* = 48), biospecimens were de-identified and unlinked to clinical data. For all cases, only non-identifiable information is presented in this publication.

### Sample collection

Tissue samples from second trimester (14–26 weeks gestational age) stillbirths, elective terminations, and spontaneous abortions were collected by the Embryo-Fetal Pathology laboratory at the B.C. Women’s Hospital and Health Centre, Vancouver, Canada. Exclusion criteria for control cases included chromosomal abnormality, congenital or brain abnormality, or grossly abnormal placenta. The use of second trimester fetal tissues required the inclusion of samples from complicated pregnancies. However, multiple pathologies were included to obtain a heterogeneous reference control group and minimize the likelihood of significant findings in association with any given pathology. For controls with known mode of fetal demise (*n* = 16), the distribution is as follows: five cases with preterm premature rupture of membranes (PPROMs), three cases of chorioamnionitis, two cases of oligohydramnios, and one case with each of: cervical incompetence, copper intrauterine device (IUD), severe IUGR, intrauterine fetal demise, spontaneous abortion, and hypoplastic left heart syndrome. In the NTD groups, chromosomally normal cases with isolated spina bifida or anencephaly were included, and the mode of termination was heterogeneous within each NTD group. Whenever possible, a sample of fetal kidney (cortex and medulla), brain (cortex), spinal cord (thoracic region or superior to the lesion in SB), muscle (psoas), and placental chorionic villi were obtained for each of 22 SB-affected fetuses, 15 AN-affected fetuses, and 19 CON fetuses (Additional file 1: Table S1); a total of 187 samples were run on the 450k array. Placental chorionic villi was obtained from an additional 9 SB, 11 AN, and 9 CON for follow-up pyrosequencing analyses (Additional file 1: Table S2). DNA was extracted from samples by standard salting out method. Our previous studies have shown that DNAm varies across the placenta [29, 30]; thus, to obtain a more accurate assessment, two independent sites (one proximal to the cord insertion and one midway between the cord and placental edge) were taken from the fetal side of each placenta and extracted DNA was combined for DNAm analyses.

### Case characteristics

Clinical case characteristics are presented in Table 1. SB and AN were compared as separate NTD status groups throughout the study, as they may have distinct etiologies [31]. Although the range of gestational ages overlapped, CON cases were younger than SB cases at delivery (median 19.0 vs. 21.8 weeks, *p* = 0.0004), and included fewer males (37 vs. 73 %, *p* = 0.03). These clinical characteristics did not differ between the AN group and CON (Table 1). A detailed list of sample information, including clinical characteristics and technical variables relating to running of the 450k array, is available in Additional file 1: Table S1.

**Table 1:** Clinical characteristics of cases

	Control (CON) *n* =19	Spina bifida (SB) *n* = 22	Anencephaly (AN) *n* = 15	*p* value^a^ (CON vs SB; CON vs AN)
Fetal GA (weeks); median (range)	19.0 (14.5–23.9)	21.8 (19.4–23.7)	20.0 (16.7–23.3)	0.0004, ns
Maternal age (years); median (range)	30.0 (21.0–41.0)	30.0 (20.1–40.5)	30.4 (22.8–37.3)	ns, ns
Fetal sex; *n* male (% male)	7 (37)	16 (73)	5 (33)	0.03, ns
Fetal *MTHFR*677 genotype; *n* (%)				ns, ns
CC	12 (63)	11 (50)	6 (40)	
CT	7 (37)	7 (32)	8 (53)	
TT	0 (0)	4 (18)	1 (7)	
Fetal *MTHFR*1298 genotype; *n* (%)				ns, ns
AA	8 (42)	14 (64)	6 (40)	
AC	9 (47)	6 (27)	8 (53)	
CC	2 (11)	2 (9)	1 (7)	
Available tissues; *n* (%)				
Chorionic villi	16 (84)	22 (100)	14 (93)	
Kidney	16 (84)	20 (91)	8 (53)	
Spinal cord	9 (47)	17 (77)	6 (40)	
Brain	11 (58)	9 (41)	–	
Muscle	13 (68)	10 (45)	8 (53)	

^a^ *p* values calculated by Mann–Whitney test for continuous variables and Fisher’s exact test for categorical variables

### *MTHFR* genotyping

*MTHFR* genotype was assessed at two loci in each case: at nucleotide 677C>T (rs1801133) and 1298A>C (rs1801131). In all individuals but one, chorionic villi was available for genotyping; in the exceptional case, kidney was used in lieu of chorionic villi. Primer sequences and reaction conditions can be found in Additional file 1: Table S3. 5 µL of PCR product was sequenced on a Pyromark Q96 MD Pyrosequencer (Qiagen) using standard protocols.

### Illumina Infinium HumanMethylation450 BeadChip (450k) quality control and pre-processing

Genomic DNA was purified and bisulfite converted as in Price et al., 2013. Samples were randomized across 3 MSA-4 plates for processing following the Illumina Infinium HumanMethylation450 BeadChip protocol [27]. Raw intensity was read into Illumina GenomeStudio Software 2011.1 and background normalization was applied.

On each array, Illumina included 835 control probes to assess, for example, array staining, extension, and bisulfite conversion. An initial quality control (QC) check following Illumina protocol was performed using the control probes, with no samples, chips, or batches identified as outliers. Signal intensity exported from GenomeStudio was read into R statistical software [32] using lumi [33] to convert signal intensities to M values. Extensive QC was conducted to check sample identity using (1) clustering of samples originating from the same individual with 65 450k SNP probes; (2) clustering of samples by sex with 450k chr X and Y probes; and (3) clustering of samples with their respective tissue using all autosomal probes. Finding no mislabeled samples, sample quality was next assessed using (1) the number of probes with a detection *p* value >0.01; (2) the number of probes with <3 bead replicates; and (3) the average sample intensity. Four samples were identified as outliers based on sample quality checks and removed from further analyses.

Probe filtering was next conducted to eliminate systemically poor quality probes (detection *p* value >0.01 in >20 % of samples or <3 bead replicates in >20 % of samples; *n* = 587), probes targeting the sex chromosomes (*n* = 11,345), polymorphic probes (*n* = 20,573), and probes that potentially cross-hybridize to the sex chromosomes (*n* = 10,672), as annotated in [34]. Color correction [33] to correct for red-green color channel bias and SWAN normalization [35] to correct for type I–type II probe bias were applied. *M* values were replaced with missing values in the remaining probe-sample pairs with detection *p* values >0.01 or <3 bead replicates.

Principal component analysis was used to detect batch effects within each tissue. MSA-4 plate, Sentrix_row (i.e., chip row), and Sentrix_ID (i.e., chip ID) were found to be associated with variability in the dataset and were subsequently corrected for using ComBat [36]. Prior to correction, an additional 244 probes were removed from all samples since there were less than two values for one of the three batch variables, resulting in 183 samples and 442,156 probes. Successive rounds of batch correction were applied starting by correcting for MSA-4 plate, followed by Sentrix_row then Sentrix_ID.

The correlation of two replicate pairs (one chorionic villi, one kidney sample) was used as a QC metric throughout pre-processing of the dataset; starting in the raw data with r_chorionic villi = 0.9953467 and r_kidney = 0.989889 and ending with r_chorionic villi = 0.9959680 and r_kidney = 0.9947478 in the batch-corrected data. With one sample of each replicate pair removed and removal of the 65 SNP probes, the final 450k array dataset included 179 samples and 442,091 “clean probes”: 52 chorionic villus samples, 44 kidney samples, 32 spinal cord samples, 20 brain samples, and 31 muscle samples (Additional file 1: Table S1).

### Probe to gene annotation

For gene-based analyses, a single gene name was annotated to each 450k CpG site in the following manner: (1) sites with no Illumina-annotated UCSC_refgene_name were annotated as NA; (2) sites with one or more gene name entries in the Illumina-annotated UCSC_refgene_name and where all gene names were identical were annotated to the given gene; (3) sites with multiple differing gene name entries in the Illumina-annotated UCSC_refgene_name, were annotated to the closest transcription start site (TSS) based on GPL16304 annotation in the Closest_TSS_gene_name column [34]. Probes that fell into category (1) were distant from TSSs. These were not annotated to a gene as the regulation of DNAm at these CpG sites may not be determined by the closest TSS.

### Differential methylation analyses

Differential methylation (DM) was assessed within a tissue by applying a linear model to *M* values on a per CpG-level using the R package limma [37]. In modeling DNAm, NTD status (CON, SB, AN) was used as the main effect and fetal sex and gestational age were included as additive covariates. DM results were extracted for the comparison of SB to CON and AN to CON. Resulting *p* values were adjusted using the Benjamini and Hochberg [38] false discovery rate (FDR) method in the limma topTable function.

*M* values corrected for fetal sex and gestational age were transformed to ß values using the m2beta function in lumi [33]. For every CpG site, average DNAm was calculated within each tissue by taking the mean *β* value for each of CON, SB, and AN, with missing data points stripped prior to calculation. Group differences in DNAm (deltaβ) were then calculated by subtracting the CON average ß from each of the SB and AN average ß per CpG site. Significant DM CpG sites were considered as those with an FDR <0.05 and deltaβ ≥0.05.

### Biologically relevant candidate CpG site analysis

DM analysis was conducted as outlined above, using only a subset of biologically relevant candidate CpG sites. Biologically relevant candidate CpG sites were chosen as those mapping to the following genes: (1) where a mutation of the homologous gene in mice has been shown to result in NTDs [39], (2) thought to be associated with human cases of NTDs [40], or (3) annotated to the GO term “one carbon metabolism process” (GO:0006730). 340 genes met one or more of these criteria, and the 8393 probes associated with these genes were used in the biologically relevant CpG site analysis (Additional file 1: Table S4).

### Genome-wide analyses–450k array

Unsupervised hierarchical clustering was performed using all clean, gestational age, and sex corrected data (*n* = 442,091 CpG sites, *n* = 179 samples). The dissimilarity structure of the data was calculated using the Euclidean method with average agglomeration. An array average DNAm was calculated for each sample by averaging DNAm at all clean CpG sites corrected for gestational age and sex (*n* = 442,091) with missing data points stripped prior to calculation. The percentage of outlier probes per sample was calculated in two steps. First, the number of outlier probes per sample was calculated within each tissue. A probe was considered an outlier if it was greater than three median absolute deviations from the probe median for all samples in the given tissue [41]. Second, the number of outlier probes per sample was normalized by dividing it by the total number of CpG sites with data (i.e., not NAs) in the given individual.

### DNA methylation by pyrosequencing

Genomic DNA from each sample was bisulfite converted using the EZ Gold DNA methylation kit (Zymo, Irvine, CA, USA), following manufacturer’s protocols. Primer sequences [42, 43] and reaction conditions for all pyrosequencing assays can be found in Additional file 1: Table S3. Within an assay, samples were randomized across pyrosequencing plates to reduce technical bias. For quality control, synthetic fully methylated and unmethylated samples (EpigenDx, Hopkinton, MA, USA) were included on each plate.

**Repetitive elements**–DNAm was averaged for four CpG sites in the LINE1 promoter and three CpG sites in Alu for assessment of repetitive element DNAm [42, 43]. Samples with a single peak height <75 or SD > 10 % between assayed CpGs were repeated.

**Chorionic villi candidate follow-up**–Four CpG sites (cg1098862, cg02413938, cg17343385, cg24666096) were identified as DM in the 450k array comparison of AN to CON. Two of these sites (cg1098862, cg02413938) were followed-up by pyrosequencing in an extended group of samples since they were close to genes which may be of biological interest in NTDs. The other two sites were not followed-up by pyrosequencing since cg17343385 overlaps a documented SNP (rs111359627, dbSNP141), which likely accounts for the observed difference in DNAm, and cg24666096 is >30 kb from the closest transcription start site. DNAm by pyrosequencing was significantly correlated with array DNAm in the set of samples run on the 450k array [cg1098862, *r* =0.84 (*p* < 2.2e−16); cg02413938, *r* = 0.37 (*p* = 0.008)].

### GO analysis

Gene ontology (GO) analysis was performed using ErmineJ [44]. For each tissue by NTD status comparison, genes associated with 450k CpG sites were ranked by magnitutde of unadjusted *p* values (smallest to largest) from the fitted linear models. Enrichment for GO classes was performed against the cleaned 450k array background using the following ErmineJ conditions: gene score resampling method, minimum gene set size of 10, maximum gene set size of 200, using the best score for gene replicates, median class scoring, 200,000 iterations, and full resampling. Gene ontology was conducted with the 748 persistent kidney spina bifida hits using the “quick list” option of the over-representation analysis (ORA) method in ErmineJ.

### DMR analysis

Differentially methylated region (DMR) analysis was conducted within a tissue on M values using the dmrFind function in the R charm package [45] with the following criteria to define a DMR: maximum gap between adjacent CpGs = 300 bps and ≥3 probes in identified DMRs. Fetal sex and gestational age were included as additive covariates in the modeling of DMRs. Once DMRs were identified in the comparison of SB to CON and AN to CON, a *q* value correction for multiple comparisons was applied with 1000 iterations using the qval function in charm.

### Publicly available data and analysis

Filtered and raw data for the 179 samples used in this publication were deposited in NCBI’s gene expression omnibus (GEO) [46] and are accessible through GEO Series accession number GSE69502 (http://www.ncbi.nlm.nih.gov/geo/query/acc.cgi?acc=GSE69502).

Additional control fetal kidney (GEO CON) data were downloaded from GEO SuperSeries, GSE30654 [47]. 450k data available for five fetal kidney samples of comparable gestational age (GSM868047, GSM868048, GSM868049, GSM868050, GSM868051) (Additional file 1: Table S5), were compared to our CON and SB kidney samples. To validate the DM kidney SB CpGs, first those CpG sites demonstrating a post hoc association with technical confounding variables were filtered out, leaving 2644 of 3342 sites for further investigation. Then, our CON and SB samples were each compared to the GEO CON samples to assess replication of DM at an FDR <0.05. CpG sites that were DM between the two control groups were removed (*n* = 1536). Finally, only those CpG sites that were DM between our SB and the GEO CON samples were considered “persistent hits” (*n* = 748 of the 2644 cleaned DM kidney SB CpGs).

### Statistical software

All analyses were conducted in R statistical software [32]. *p* values for Tables 1, 2 were calculated by Mann–Whitney test for continuous variables and Fisher’s exact test for categorical variables. For chorionic villi AN follow-up analyses, correlation of pyrosequencing and array data was conducted using Spearman’s rank order correlation. A linear model with main effect NTD status and fetal sex and gestational age as additive covariates was fit to each follow-up CpG site. Correction for multiple comparisons was applied using Bonferroni correction. Graphics were created using the ggplot2 package [48].

**Table 2:** Genome-wide DNA methylation by tissue

		Chorionic villi	Kidney	Spinal cord	Brain	Muscle
Median array average ± SD (*n*)	Control	0.432 ± 0.006 (16)	0.471 ± 0.004 (16)	0.486 ± 0.002 (9)	0.486 ± 0.004 (11)	0.484 ± 0.004 (13)
	Spina bifida	0.427 ± 0.007 (22)**	0.470 ± 0.003 (20)	0.481 ± 0.004 (17)*	0.482 ± 0.004 (9)	0.484 ± 0.003 (10)
	Anencephaly	0.425 ±0.007 (14)*	0.468 ± 0.003 (8)	0.485 ± 0.003 (6)	–	0.484 ±0.003 (8)
Median % LINE1 DNAm ± SD (*n*)	Control	55.8 ± 3.8 (16)	79.2 ± 1.5 (16)	79.9 ± 1.5 (9)	81.5 ± 2.0 (11)	80.3 ± 1.4 (13)
	Spina bifida	56.7 ± 3.0 (22)	78.3 ± 2.1 (20)	80.9 ±1.7 (17)	83.2 ± 1.4 (9)	80.9 ± 1.6 (10)
	Anencephaly	55.0 ± 2.9 (14)	78.5 ± 1.4 (8)	79.7 ± 1.7 (6)	–	80.5 ± 1.4 (8)
Median % Alu DNAm ± SD (*n*)	Control	22.6 ±1.1 (16)	24.7 ± 1.2 (16)	25.0 ± 0.9 (9)	25.6 ± 1.6 (11)	24.2 ±1.0 (13)
	Spina bifida	22.3 ± 1.1 (22)	24.8 ± 1.5 (20)	26.1 ± 1.6 (17)	25.4 ± 1.2 (9)	25.0 ± 2.2 (10)
	Anencephaly	22.8 ± 1.3 (14)	24.1 ± 2.1 (8)	25.4 ± 0.9 (6)	–	24.5 ± 0.6 (8)

*p* values based on comparison of spina bifida to controls or anencephaly to controls. *p < 0.05 based on 1000 permutations of Mann–Whitney test, **p < 0.01 based on 1000 permutations of Mann–Whitney test. *SD* standard deviation

## Results

### *MTHFR* genotyping

Two SNPs in *MTHFR*, 677C>T, and 1298A>C, were evaluated in our cases as (1) reduced MTHFR function has been reported for carriers of 677TT (~30 % function of controls) and 1298CC (~57 % function of controls) [10, 11]; and (2) an association between the 677TT genotype and NTD status has been consistently reported (odds ratio of 1.8 for NTD infant carriers) [9]. In our small population, no difference in genotype frequency by NTD status was observed at the 1298 locus. A trend for increased T homozygotes at the 677 locus was present in both NTD groups compared to CON, although this did not reach statistical significance (Table 1). Given our small sample size, we did not evaluate additional genetic polymorphisms.

### Differential methylation of biologically relevant candidate CpG sites

Specific disease-relevant loci may play a role in the development of NTDs through altered gene expression. These include genes for which mutations in mice lead to the development of NTDs, in addition to loci involved in one-carbon metabolism. As DNAm can either affect or reflect gene expression, we first assessed DNAm at 8393 candidate 450k CpG sites in 340 biologically relevant genes (Additional file 1: Table S4). Within each tissue, a linear model was fit for every biologically relevant CpG site to test for differential methylation by NTD status, while controlling for fetal sex and gestational age. Differentially methylated (DM) CpG sites were those identified at a false discovery rate (FDR) <0.05 and average group difference in DNAm (deltaβ) ≥0.05, when comparing SB to CON or AN to CON (see “Methods”). DM CpG sites were detected only in the kidney comparison of SB to CON (*n* = 65, 0.8 % of biologically relevant candidate CpG sites), while comparisons in all other tissues did not meet the DM criteria (Additional file 1: Table S6). We conclude that aberrant DNAm of these biologically relevant candidate loci is not a main feature of our NTD cases, with the exception of SB kidney.

### Genome-wide DNA methylation

**Fig. 1:**
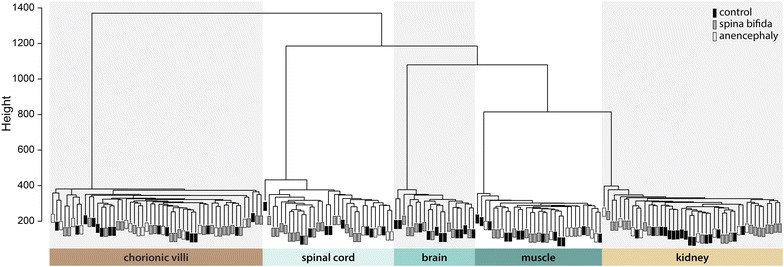
Sample clustering based on array-wide DNA methylation. Unsupervised hierarchical clustering of 442,091 CpG sites clustered samples primarily by tissue type. Within a tissue, samples did not cluster by NTD status. We did note however, a cluster of eight spina bifida and one anencephaly in kidney and a cluster of 12 spina bifida and two controls in spinal cord

Demand for one carbon units is high during times of rapid cell proliferation, development, and migration [12], such as in early embryo development. If OCM cycling is disrupted in NTD cases persisting in British Columbia, we expected that widespread changes in DNAm would be observed. We took several approaches to address this question. First, unsupervised hierarchical clustering of clean 450k CpG sites (*n* = 442,091 CpGs, see “Methods”) gave an overall view of the relationship between study samples (Fig. 1). As expected, samples clustered primarily by tissue type, not the individual of origin. Tissues originating from the inner cell mass clustered closer together (i.e., brain and spinal cord of ectodermal origin; muscle and kidney of mesodermal origin), while chorionic villi (of mixed trophectodermal/chorionic mesodermal origin) clustered further away from the four somatic tissues. Notably, even within a tissue, there was no clear division of samples by NTD status, although we noted a cluster of eight SB and one AN in kidney, and of twelve SB and two CON in spinal cord (Fig. 1).

All cases of NTDs might not exhibit aberrant genome-wide DNAm; their multifactorial inheritance suggests that the etiology of this disease is heterogeneous. Thus second, we calculated both (1) the 450k array average DNAm per sample (Table 2; Additional file 2: Fig. S1) and (2) the percentage of outlying CpG sites per sample (Additional file 2: Fig. S2). Using 1000 permutations of the Mann–Whitney test, a slight reduction in array average DNAm was noted in chorionic villi for SB vs. CON (−0.005, *p* < 0.01) and AN vs. CON (−0.007, *p* < 0.05; Table 2). Comparing between NTD status groups, there was no difference in the number of outlier samples based on either of these measures.

Finally, a different assay was used to measure global DNAm in all samples—pyrosequencing of the repetitive elements LINE1 and Alu [42, 43]. LINE1 and Alu repetitive elements are scattered throughout the genome and their DNAm has been shown to be sensitive to environmental changes [49–52]. These repetitive elements are not densely covered by the 450k array and thus, they were used to assess DNAm at other genomic regions. There was no statistical difference in repetitive element DNAm by NTD status in any of the studied tissues (Table 2).

### Differential methylation of CpG sites array-wide

**Fig. 2:**
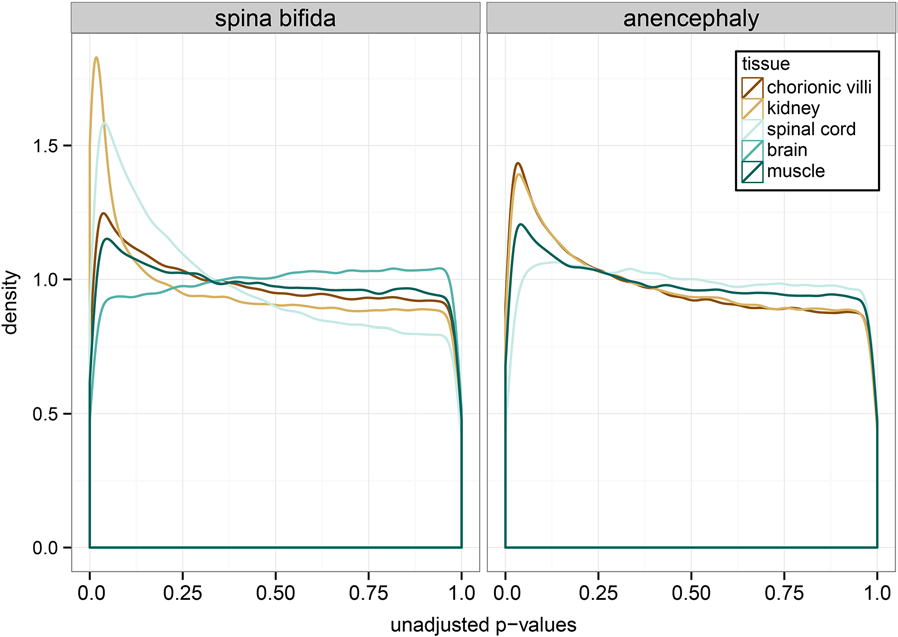
Tissue distribution of unadjusted *p* values from linear modeling of differential methylation in NTDs. Distribution of *p* values from the comparison of DNA methylation for spina bifida to control samples at each of 442,091 CpG sites (*left*) and for anencephaly to controls samples at each of 442,091 CpG sites (*right*), including fetal gestational age and sex as additive covariates. *Flat* distributions indicate equal likelihood of significant and non-significant tests, while *left-peaking* distributions indicate greater likelihood of significant tests

We next explored whether specific CpG sites with unknown relationship to NTDs exhibited altered DNAm by testing for differential methylation (DM) at each 450k array target. Within a tissue, a linear model was fit per CpG site to test for DM by NTD status while controlling for fetal sex and gestational age. The distribution of unadjusted *p* values from the comparisons of SB and AN to CON cases gives a broad view of the pattern of DM by NTD status (Fig. 2). A uniform distribution of *p* values, for example, in the AN spinal cord comparison, indicates equal likelihood of DM and non-DM. Left-peaking distributions (e.g., SB kidney or spinal cord comparisons), indicate a greater likelihood of DM than non-DM, suggesting that a subset of loci show altered DNAm in these tissues. These differences, however, must pass correction for multiple comparisons to be considered significant CpGs of interest.

**Fig. 3:**
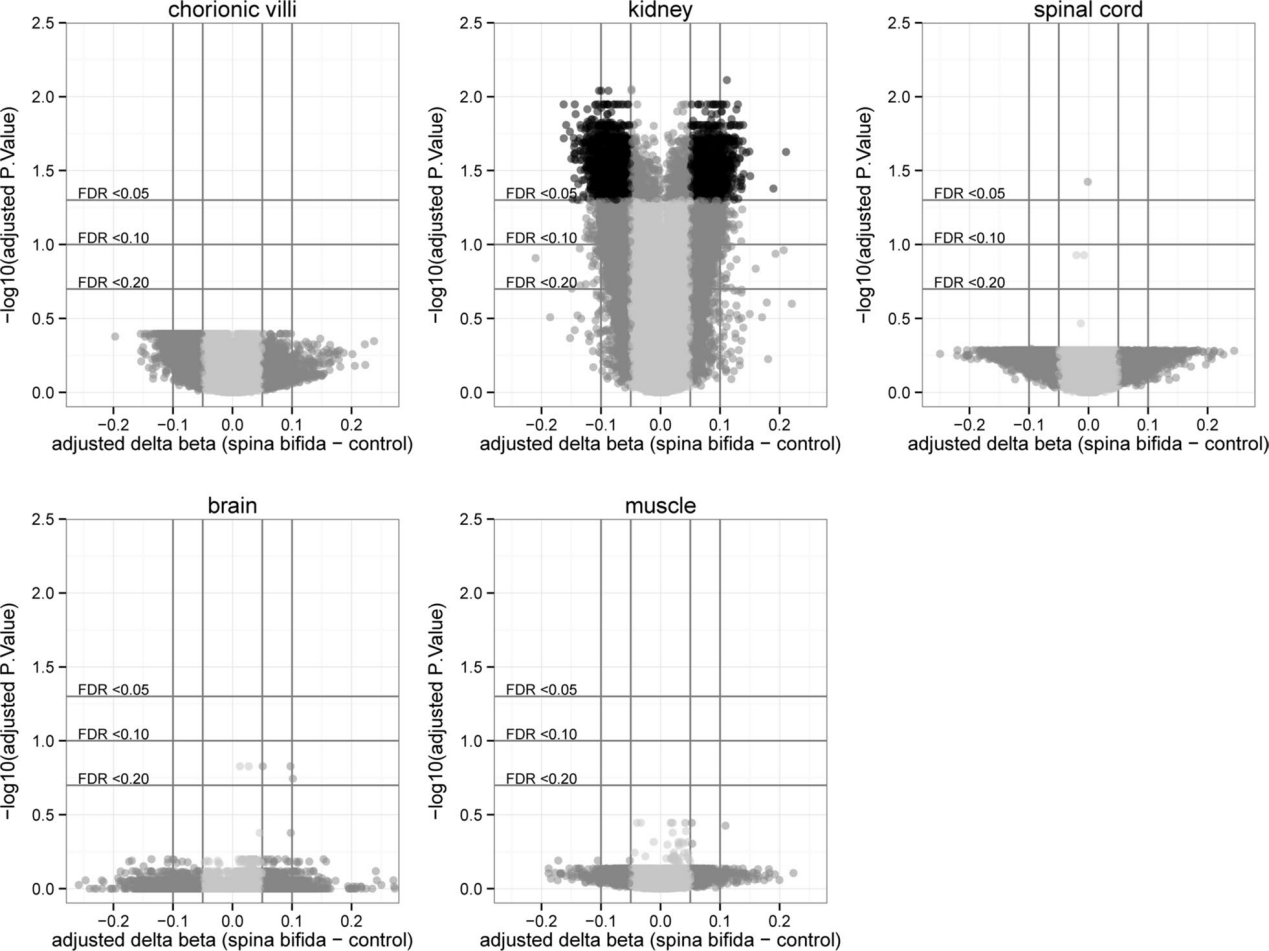
Spina bifida array-wide volcano plots. Volcano plots comparing the magnitude of difference in DNAm (adjusted delta beta) to statistical significance (−log10(adjusted P.Value)) for each CpG site (*n* = 442,091) in spina bifida vs. control samples

**Fig. 4:**
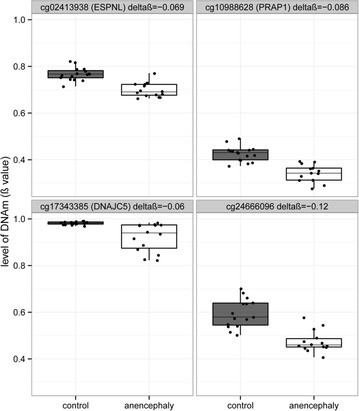
Differentially methylated CpG sites in the chorionic villi comparison of anencephaly cases to controls. Four CpG sites were identified as significantly differentially methylated at an FDR <0.05 and deltaβ ≥0.05. Each plot is labeled with the 450k CpG site identifier, the gene which it is closest to and average difference in DNAm between anencephaly and controls (deltaβ). *Box edges* are plotted at the 25th and 75th percentiles (the inter-quartile range (IQR)) and whiskers are plotted to the last sample within ± 1.5*IQR

An FDR correction was applied to the *p* values obtained from the fitted linear models; additionally, for every CpG site, the average CON DNAm was subtracted from the average SB or AN DNAm (deltaβ). Plotting deltaβ against FDR for each CpG site demonstrated remarkably few extreme differences in most of the tissue comparisons, except for the comparison of SB kidneys to CON kidneys (Fig. 3; Additional file 2: Fig. S3). The deltaβ on the x-axis of these volcano plots (Fig. 3) suggests that the array-wide difference in average DNAm noted in chorionic villi in the previous section (Table 2) is representative of small changes in DNAm across many CpGs. At a statistical threshold of FDR <0.05, 4148 CpG sites were DM in the kidney comparison of SB to CON, one CpG site was DM in the spinal cord comparison of SB to CON, and five CpG sites were DM in the chorionic villi comparison of AN to CON (Additional file 1: Table S7). The application of a second filter, a minimum deltaβ of 0.05 to enhance for biologically meaningful differences between groups, reduced the number of DM CpG sites to 3342 in the kidney comparison of SB to CON and four (Fig. 4) in the chorionic villi comparison of AN to CON (discussed in next section).

Four approaches were taken to reduce the risk that findings from the above DM analysis were not limited due to threshold cutoffs, sample size, or small differences in DNAm. First, the top 1000 ranking DM CpG sites were overlapped (1) within a tissue by NTD status (Additional file 2: Fig. S4), and (2) within an NTD status across all tissues (Additional file 2: Fig. S5). There was little overlap of top ranking DM CpG sites in either comparison.

Second, for each tissue and NTD status comparison, a gene ontology (GO) analysis was conducted with all unadjusted *p* values to rank genes associated with CpG sites on the array. The only GO classes significant at an FDR <0.10 were “nuclear-transcribe mRNA catabolic process, nonsense-mediated decay” (GO:0000184, 111 genes) in the chorionic villi SB to CON comparison and “production of molecular mediator involved in inflammatory response” (GO:0002532, 13 genes) in the spinal cord AN to CON comparison.

Third, we assessed DM regions (DMRs) rather than DM CpG sites to integrate information from neighboring CpG sites and reduce the number of comparisons in statistical tests. DMRs were identified in each of the tissue by NTD status comparisons, but after correction for multiple comparisons, none were significant.

Finally, a subgroup analysis was performed to confirm that unequal group gestational ages (Table 1) did not obscure differential DNAm between SB and CON comparisons in chorionic villi, brain, spinal cord, or muscle. With the exception of four sites in chorionic villi, no significant differences in DNAm were noted between the gestational age-matched AN and CON groups. This allowed the AN and CON cases to be combined into a “non-SB” group, from which 19 samples (19–24 weeks) were selected for a gestational age-matched comparison to SB (Additional file 2: Fig. S6). DM between the non-SB and SB groups was tested by fitting a linear model within each tissue for NTD status with gestational age and sex as additive covariates. No CpG sites were DM at FDR <0.05 in this supplemental gestational age-matched comparison, thus differences in gestational age between SB and CON groups do not likely contribute to the lack of statistical difference observed in most of the SB tissues. These supplemental analyses, in addition to the array-wide volcano plots (Fig. 3; Additional file 2: Fig. S3), suggest that aberrant genome-wide DNAm is present in SB kidneys, but may not be a major feature of the other NTD tissues assessed in this study.

### Follow-up of differentially methylated CpG sites in kidney and chorionic villi

**Fig. 5:**
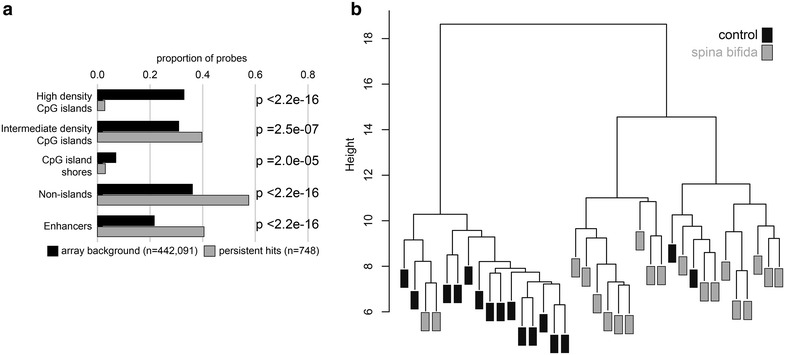
Identification and investigation of persistent hits in spina bifida kidneys. **a** By comparing our spina bifida (*SB*) and control (*CON*) samples to an independent control group (*n* = 5, GEO CON), 748 of the differentially methylated kidney spina bifida CpG sites were identified as persistent hits. Persistent hits were enriched for CpG sites located in enhancers and outside of CpG islands. **b** Hierarchical clustering of our spina bifida and control samples based solely on persistent hits almost completely separated the spina bifida from control cases, and two groups of SB cases emerged

To confirm the unique findings in kidney SB, we compared our SB cases and CON to 450k data from five independent control second trimester fetal kidney samples downloaded from GEO (GEO CON; Additional file 1: Table S5). Of the 4148 CpG sites identified as DM in the previous section at FDR <0.05, 2644 (64 %) remained DM at FDR <0.05 when comparing our SB kidneys to the GEO CON kidneys. A subset of 748 higher confidence CpG sites, termed persistent hits, was selected for use in follow-up analyses in SB kidneys. Persistent hits were refined using four filters: (1) CpG sites identified as DM at FDR <0.05 and deltaβ ≥0.05 in the previous section when comparing our SB to CON kidneys (3342 of 4148); (2) CpG sites where a post hoc test revealed no association with study covariates—gestational age, sex, plate, chip, or row (2644 of 3342); (3) CpG sites where the two control groups exhibited no DM at FDR <0.05 (1536 of 2644); and (4) CpG sites with DM at FDR <0.05 and deltaβ ≥0.05 between our SB and the GEO CON samples (748 of 1536). Persistent hits were under-represented in high-density CpG islands (*p* < 2.2e–16) and enriched in non-islands (*p* < 2.2e–16) and enhancers (*p* < 2.2e-16) (Fig. 5a), and were not enriched for any GO terms. As expected, supervised clustering of our kidney samples using only these 748 persistent hits almost entirely separated the SB from CON cases, though interestingly, two distinct clusters emerged within the SB cases (Fig. 5b). These two SB clusters, however, did not separate by sex, gestational age, location of the defect, or *MTHFR* genotype. The persistent hits were not followed-up as they appear to be secondary changes, and are not likely linked to our etiology of NTD hypothesis.

Four sites (cg1098862, cg02413938, cg17343385, cg24666096) were identified as DM in the AN vs. CON comparison in chorionic villi (Fig. 4). Pyrosequencing assays (Additional file 1: Table S3) were designed to follow up two of these CpG sites using this independent technique in the chorionic villus samples run on the 450k array (*n* = 16 CON, 22 SB, 14 AN) in addition to an extended set of chorionic villi samples (*n* = 9 CON, 9 SB, 11 AN; Additional file 1: Table S2). Follow-up of cg17343385 was excluded as a post hoc search of UCSC genome browser indicated that this probe targeted a SNP not excluded in the filtering steps of data processing. In the case of cg24666096, follow-up was not conducted as no nearby functional genomic elements were annotated in UCSC genome browser (i.e., TF binding sites, TSS, or ENCODE enhancers) making it currently difficult to interpret the biological relevance of altered DNAm at such a location. The difference in DNAm at cg10988628, 146 bps upstream of *PARP1,* was validated by pyrosequencing in the samples run on the array (*p* = 0.000006, Additional file 2: Fig. S7) and replicated in the extended samples (*p* = 0.02). The difference in DNAm observed at cg02413938, 21 bps upstream of *ESPNL,* was also validated by pyrosequencing in the samples run on the array (*p* = 0.05), although it was not replicated in the extended samples (Additional file 2: Fig. S8A). DNAm at this CpG site may be linked to a SNP 6 bps downstream (rs6431579), and it is therefore possible that cg02413938 was picked up in the 450k DM analysis due to unequal genotype distribution between NTD status groups (Additional file 2: Fig S8B).

## Discussion

Increased methyl group availability is one of the proposed mechanisms for how folic acid (FA) fortification contributed to the worldwide reduction of NTDs. We profiled DNA methylation (DNAm) in five fetal tissues ascertained from cases of NTDs in British Columbia, a folate replete population, to test whether abnormal DNAm was a feature of NTDs that develop despite FA fortification. Barring distinctive DNAm in the kidneys of spina bifida (SB) cases, neither aberrant CpG site-specific nor genome-wide DNAm were characteristic of the tissues in this cohort. Aside from our main findings, there were remarkably few changes in DNAm in anencephalic (AN) fetuses, which suggests that normal fetal neural function may not be required for placental and fetal development through the second trimester.

Though tissues directly involved in the neural tube defect—spinal cord and brain—had largely normal DNAm in NTDs, a tissue peripheral to the defect—kidney—was the one dramatic exception to this pattern, with 4148 CpG sites (FDR < 0.05) identified as differentially methylated (DM) sites in spina bifida (SB). This distinct pattern may be reflective of cell type heterogeneity in kidney, consistency of tissue sampling and/or true differences in DNAm, and warranted further investigation. When compared to a publically available control kidney group, 64 % of the kidney SB vs. CON sites remained differentially methylated, increasing confidence in this distinctive DNAm profile. However, this was not an ideal validation, as an independent SB group was not also available.

About 50 % of people living with SB exhibit abnormal urodynamics, which in many cases leads to severe renal complications [53]. However, only about 8–9 % of SB cases present with a congenital renal abnormality at birth, including for example renal agenesis, horseshoe kidney, or ureteral duplication [54, 55]. All of the 20 SB cases in this study were found to have histologically normal kidneys on autopsy, and thus grossly abnormal renal morphology does not likely account for the differential methylation we observed. Caudal neural tube closure should occur at around day 28 of gestation and the ureteric buds, which induce differentiation of the kidneys, develop at about day 30–32 [54]. Thus, an environmental disruption during this window of pregnancy leading to the development of an NTD might also be related to the abnormal kidney DNAm observed here [53]. Alternatively, this abnormal pattern of DNAm might be related to dysregulated innervation from the spinal cord during renal development. Sympathetic renal innervation originates from the T10 to L1 regions of the spinal cord [56] and is posited to play a role in the cellular and biochemical development of this organ during gestation [57]. Of our 20 SB kidneys, the highest intact level of the spinal cord was known for 15 cases: above T10—2 cases, T10-L1 region—3 cases, L2-L3—7 cases, L4-L5—2 cases, and S1—1 case. Given the distribution of location of defects in our cases and the lack of biological pathway enrichment in the persistent DM kidney CpGs, we suggest that the abnormal kidney DNAm does not likely give rise to the NTD.

Placental chorionic villi was a tissue we predicted might be more likely to exhibit changes in DNAm in NTDs. Placental DNAm may be particularly sensitive to the in utero environment [58], since this organ is designed to respond to fetal, as well as maternal, signals. The placenta is also responsible for nutrient acquisition from maternal circulation during gestation. Decreased placental weight and increased placental immaturity has been reported in anencephalic pregnancies at term [59, 60]. There was a weak trend for differential methylation in several of our chorionic villi analyses, though overall, we noted relatively little significant change in NTDs. One of the significant DM sites we identified (cg10988628) may be of interest in NTDs, since it is located 146 bps upstream of poly (ADP-Ribose) Polymerase 1 (*PARP1*). This gene is known to regulate trophoblast differentiation [61], and is involved in ADP-ribosylation of histones [62], and its loss of function is associated with invasive and metastatic properties in cancer [63]. However, the changes we noted were subtle in AN-associated placentas, and would require a larger sample size to characterize.

To our knowledge, there has been one other study of genome-wide DNAm in NTD placenta, which used the 450k array to compare DNAm of eight normal (40 weeks gestational age) to eight SB (27–40 weeks gestational age) placentas [64]. The authors identified 3839 DM sites at a *p* value = 0.01 and fold change = 0.2. A stricter threshold for DM was used in the current study, FDR <0.05 and deltaβ ≥0.05, as is recommended for EWAS to reduce false positive results [65]. Using these same criteria, we previously observed dramatic differences in the DNAm of chorionic villi in other, less severe conditions such as preeclampsia (30,248 CpG sites), and trisomy confined to the placenta (24,621 CpG sites) [66, 67], versus only four sites identified here in AN and none in SB. The difference in results between Zhang et al. [64] and the current study is likely related to use of different thresholds for significance, correction for multiple comparisons, smaller sample size, and non-overlapping gestational age of cases and controls.

Our ability to detect significant differences at individual CpGs in any of the tissues studied here may have been limited by (1) large variation in DNAm within a group, (2) pathological heterogeneity of the CON group, (3) between group differences in gestation age, (4) small average group differences in DNAm, and/or (5) sample size given 1–4. We also did not have access to important clinical information such as use of periconceptional folic acid or maternal ethnicity, though ethnic differences between cases and controls are expected to lead to false positives and are unlikely to cause false negatives. Furthermore, since the same individuals contributed multiple tissues to the study, if mismatched ethnicity was driving these differences in DNAm, we would have seen overlap of top-ranking sites across tissues (Additional file 2: Figure S5). As evidenced by the findings in SB kidneys, our study, despite these limitations was sufficient in size to indicate that large and widespread differences in DNAm are not a consistent feature of nervous or placental tissues from NTDs in British Columbia, Canada. However, an independent study in a larger cohort, covering CpG sites beyond those targeted by the 450k microarray, will be needed to definitively determine if small alterations in DNAm at specific sites are yet another contributing factor in the multifactorial development of NTDs.

## Conclusions

Most studies of pregnancy are limited to sampling placenta, cord blood, amniotic fluid, or extraembryonic membranes; tissues which may have limited applicability to the disease in question. In this study of neural tube defect (NTD) cases and controls, the Illumina 450k platform was used to profile DNA methylation (DNAm) at more than 450,000 biologically relevant CpG sites across the genome in placenta (chorionic villi), kidney, spinal cord, brain, and muscle tissue obtained from 56 fetuses. The use of this unique set of tissues increases biological relevance, and the data provide a novel resource for those studying DNAm during fetal development. We found aberrant array-wide DNAm in the kidneys of spina bifida cases, but this was not apparent in other spina bifida or anencephaly-associated tissues. Examination of the DNAm profile of organs with spinal innervation originating below that of the kidney, such as the bladder, may provide further evidence for or against our hypothesis for the observed differential methylation in spina bifida kidneys. The findings of this study were inconsistent with a generalized phenomenon of altered DNAm in NTDs that persist in folate replete populations, though we cannot exclude the DNAm hypothesis in other circumstances (i.e., nutritional environment, demographics, tissues) or regions of the genome not assessed by this study. Other mechanisms, such as changes in non-DNAm regulation of gene expression, altered cell cycle length, or deficiency of vitamins involved in one-carbon metabolism are areas of investigation that may provide further clues as to the etiology of NTDs that persist in folate replete populations.

## Consent for publication

Not applicable.

## Additional files

10.1186/s13072-016-0054-8 **Table S1** is a table listing the clinical and technical variables for the tissue samples used in the 450k microarray analysis. **Table S2** is a table listing the samples used in follow-up pyrosequencing in chorionic villi. **Table S3** is a table listing conditions used for each pyrosequencing assay. **Table S4** is a table listing probes used in the candidate gene 450k analysis. **Table S5** is a table listing the additional control kidney samples downloaded from GEO. **Table S6** is a table summarizing the results of the candidate gene differential methylation analysis. **Table S7** is a table summarizing the results of the array-wide differential methylation analysis.
10.1186/s13072-016-0054-8 **Fig. S1** is a box plot of array-wide average DNAm per sample. **Fig. S2** is a box plot of the percentage of outlier CpG sites per sample. **Fig. S3** are volcano plots of DM between anencephaly and controls at cleaned 450k CpG sites. **Fig. S4** is a Venn diagram overlapping the top 1000 DM CpG sites by NTD status for each tissue. **Fig. S5** is a Venn diagram overlapping the top 1000 DM CpG sites by tissue for each NTD status. **Fig. S6** is a plot of the cases used in the gestational age-matched non-SB vs. SB comparison. **Fig. S7** is a box plot of DNAm in chorionic villi measured by pyrosequencing in the follow-up of cg1098862. **Fig. S8** is a box plot of DNAm in chorionic villi measured by pyrosequencing in the follow-up of cg02413938.

## Abbreviations

450k array: Illumina Infinium HumanMethylation450 BeadChip
AN: anencephaly
chr: chromosome
CON: control
deltaβ/delta beta: difference in average DNA methylation
DM: differentially methylated or differential methylation
DMR: differentially methylated region
DNAm: DNA methylation
FA: folic acid
FDR: false discovery rate
GEO: gene expression omnibus
GEO CON: control kidney samples downloaded from GEO
GO: gene ontology
MTHFR: methylenetetrahydrofolate reductase
ns: not significant
NTD: neural tube defect
OCM: one carbon metabolism
QC: quality control
SB: spina bifida
SD: standard deviation
wks: weeks
yrs: years
