# Supplementary material for: Profiling placental and fetal DNA methylation in human neural tube defects
Source: Epigenetics Chromatin. 2016 Feb 16;9:6. doi: 10.1186/s13072-016-0054-8 (PMC4756451; doi:10.1186/s13072-016-0054-8)

**Additional file 2**

**Profiling Placental and Fetal DNA Methylation in Human Neural Tube Defects**

E Magda Price, Maria S Peñaherrera, Elodie Portales-Casamar, Paul Pavlidis, Margot I Van Allen, Deborah E McFadden and Wendy P Robinson*

**Fig.S1 Array average DNA methylation.** Box plots of array average DNAm (n=442,091 CpG sites) calculated for each sample and plotted by NTD status. Box edges are plotted at the 25^th^ and 75^th^ percentiles (the inter-quartile range (IQR)), whiskers are plotted to the last sample within +/- 1.5*IQR, samples beyond whiskers are outliers plotted as points. There was a small, but significant difference in average DNAm (avgβ) in chorionic villi between the SB and CON (*p*<0.01) and AN and CON (*p*<0.05) in addition to spinal cord in SB compared to CON (*p*<0.05) (also see Table 2).

**
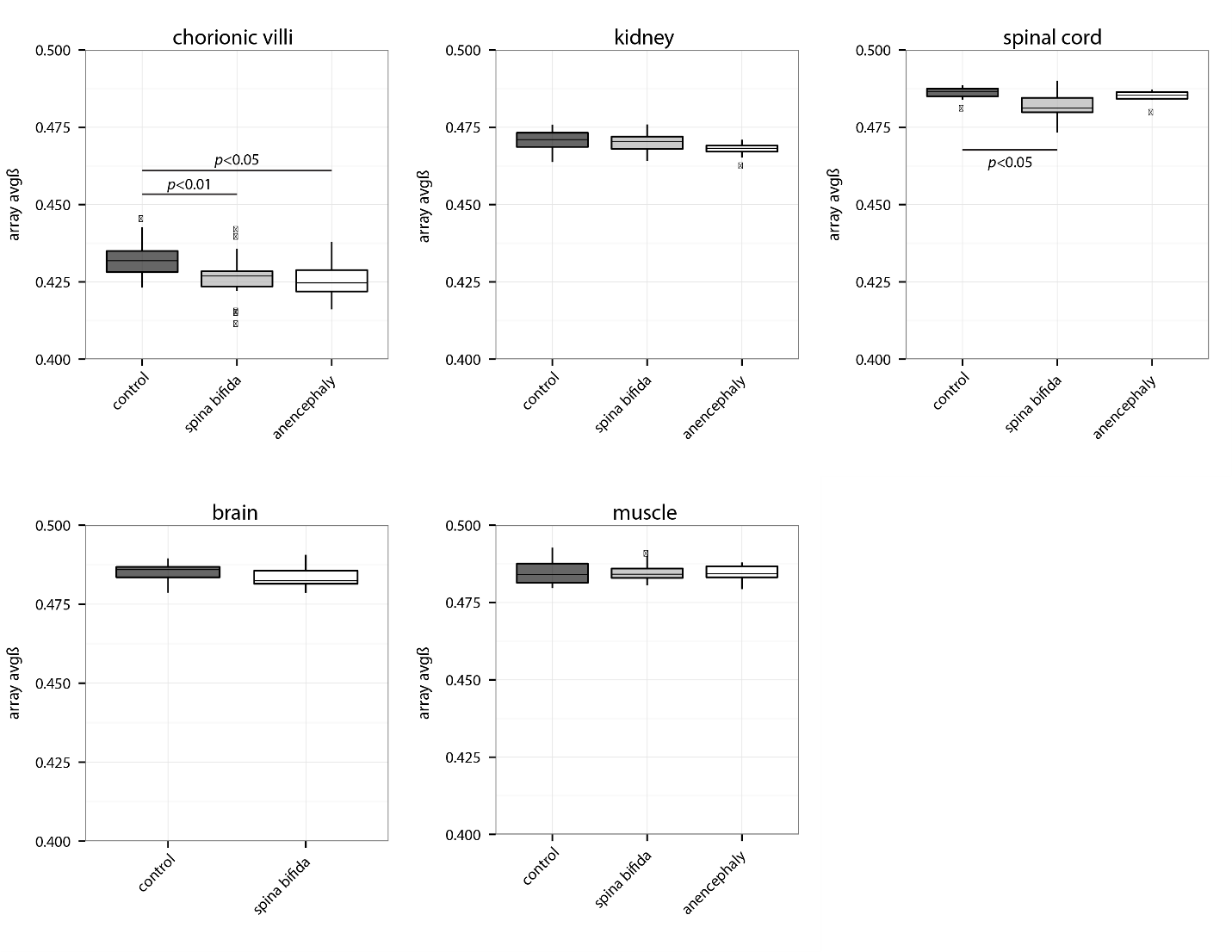
**

**Fig.S2** **Percentage of outlier CpG sites.** Box plots of percentage of outlier CpG sites (i.e. 450k array probes) calculated for each sample and plotted by NTD status. A CpG site-sample combination with >3 median absolute deviations from the site median for all samples in that tissue was called an outlier. The number of outlier probes per sample was normalized by dividing by the number of CpG sites with data for that sample (as the number of missing values varied by sample). Box edges are plotted at the 25^th^ and 75^th^ percentiles (the inter-quartile range (IQR)), whiskers are plotted to the last sample within +/- 1.5*IQR, samples beyond whiskers are outliers plotted as points. There was no difference in the percentage of outlier probes/sample by NTD status.


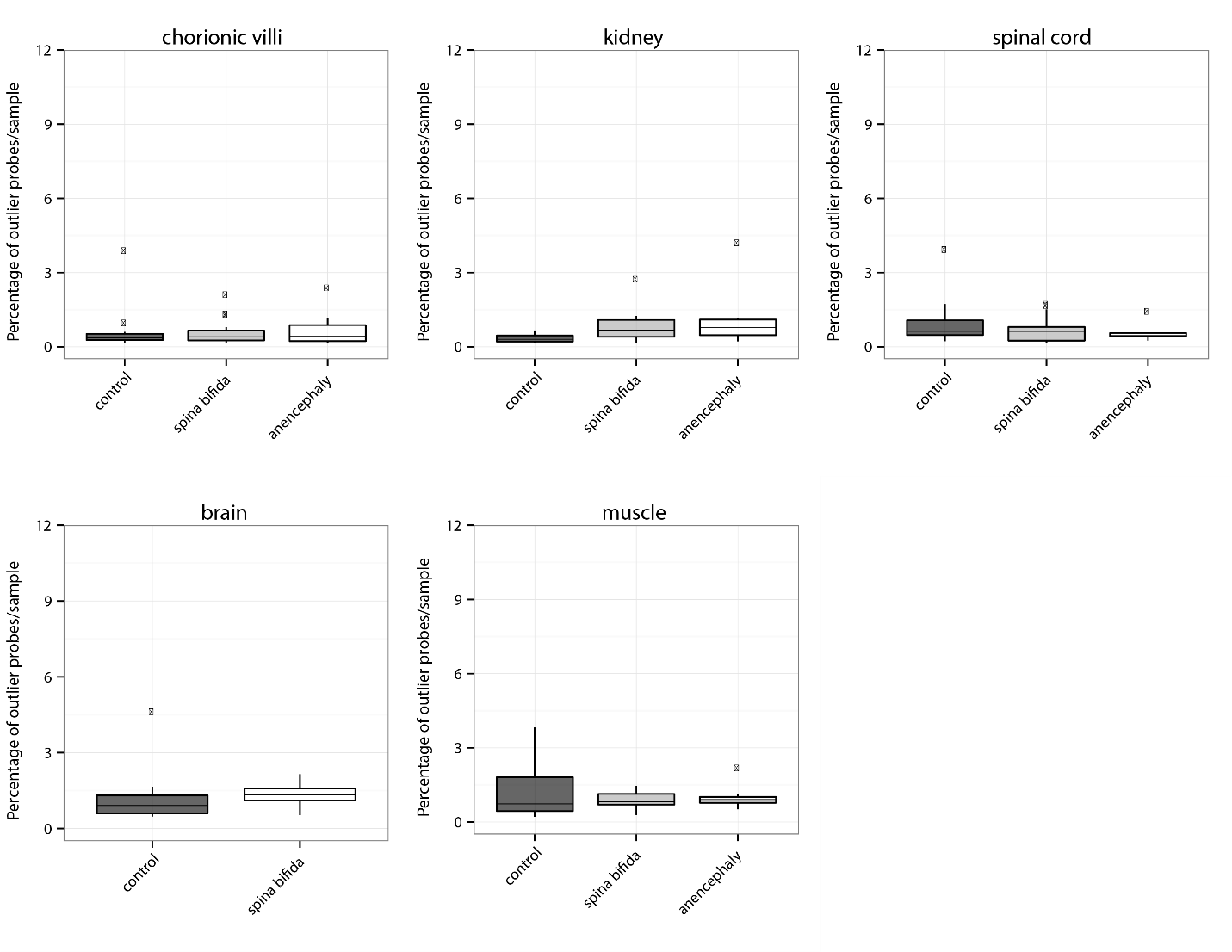


**Fig.S3 Anencephaly array-wide volcano plots.** Volcano plots comparing the magnitude of difference in DNAm (adjusted delta beta) to the statistical significance (-log10(adjusted P.Value)) for each CpG site (n=442,091) in anencephaly *vs.* control samples.


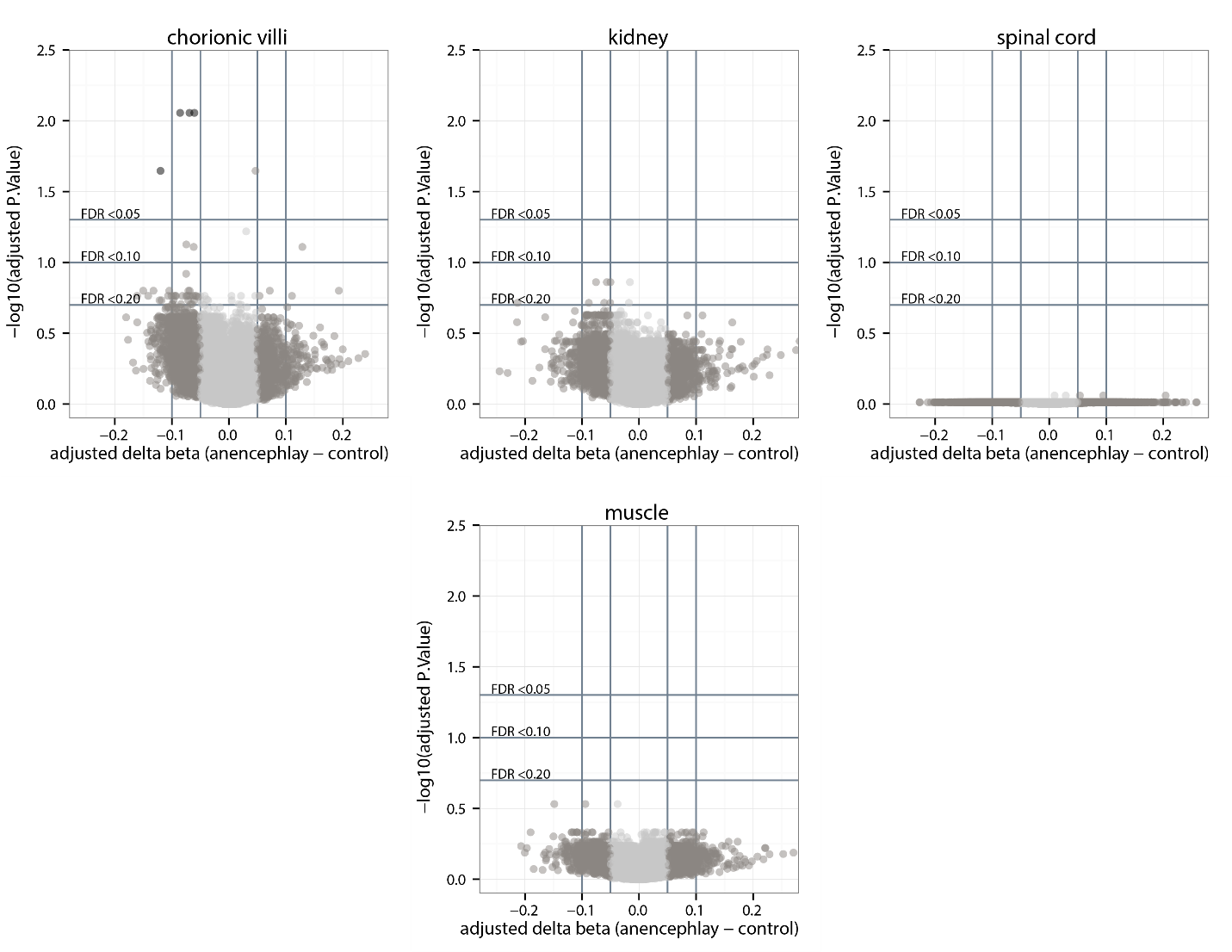


**Fig.S4** **NTD status overlap of top 1,000 CpG sites by tissue.** Venn diagram overlapping the top 1,000 ranking CpG sites for each tissue based on unadjusted *p*-values for the comparison of SB to CON and AN to CON. Brain tissue could not be included in this analysis as no brain could be collected from AN cases.


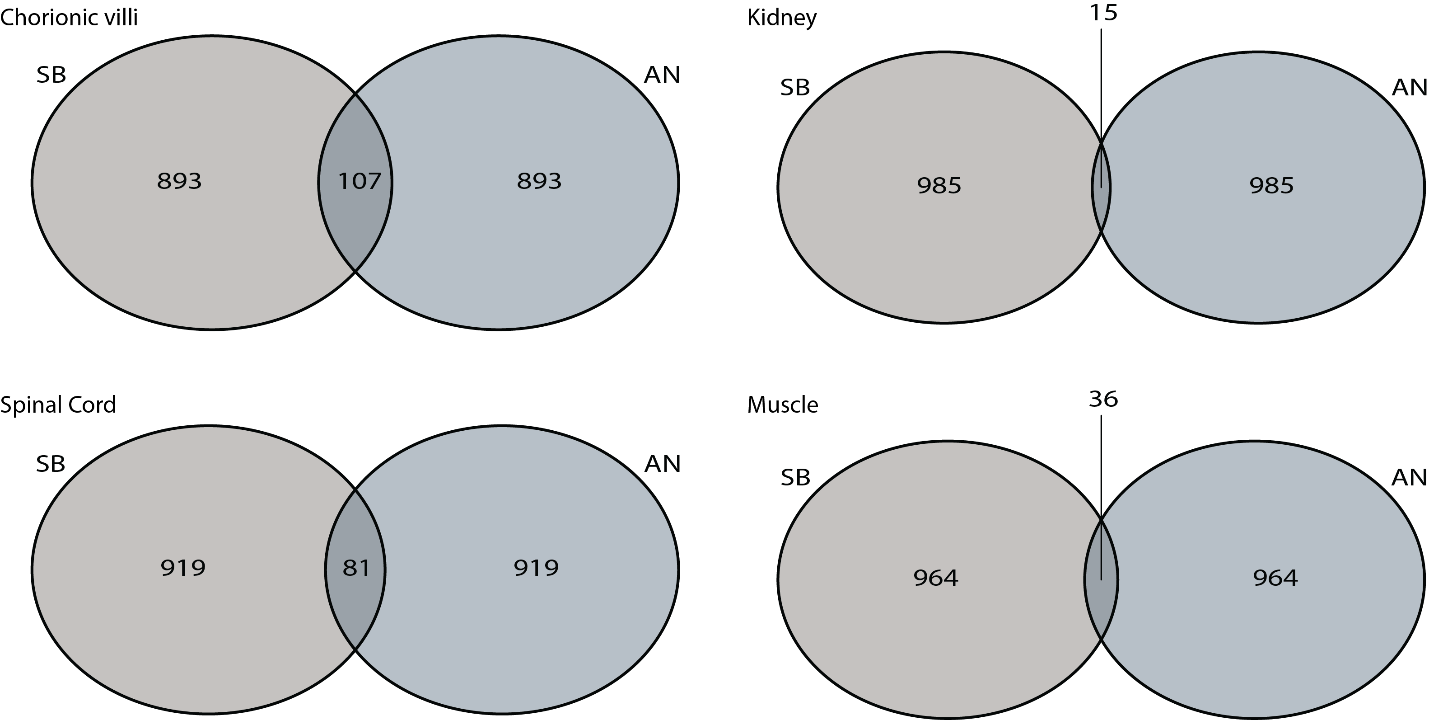


**Fig.S5 Tissue overlap of top 1,000 CpG sites by NTD status.** Venn diagram overlapping the top 1,000 ranking CpG sites across tissues within an NTD status, based on unadjusted *p*-values for the comparison of SB to CON and AN to CON.


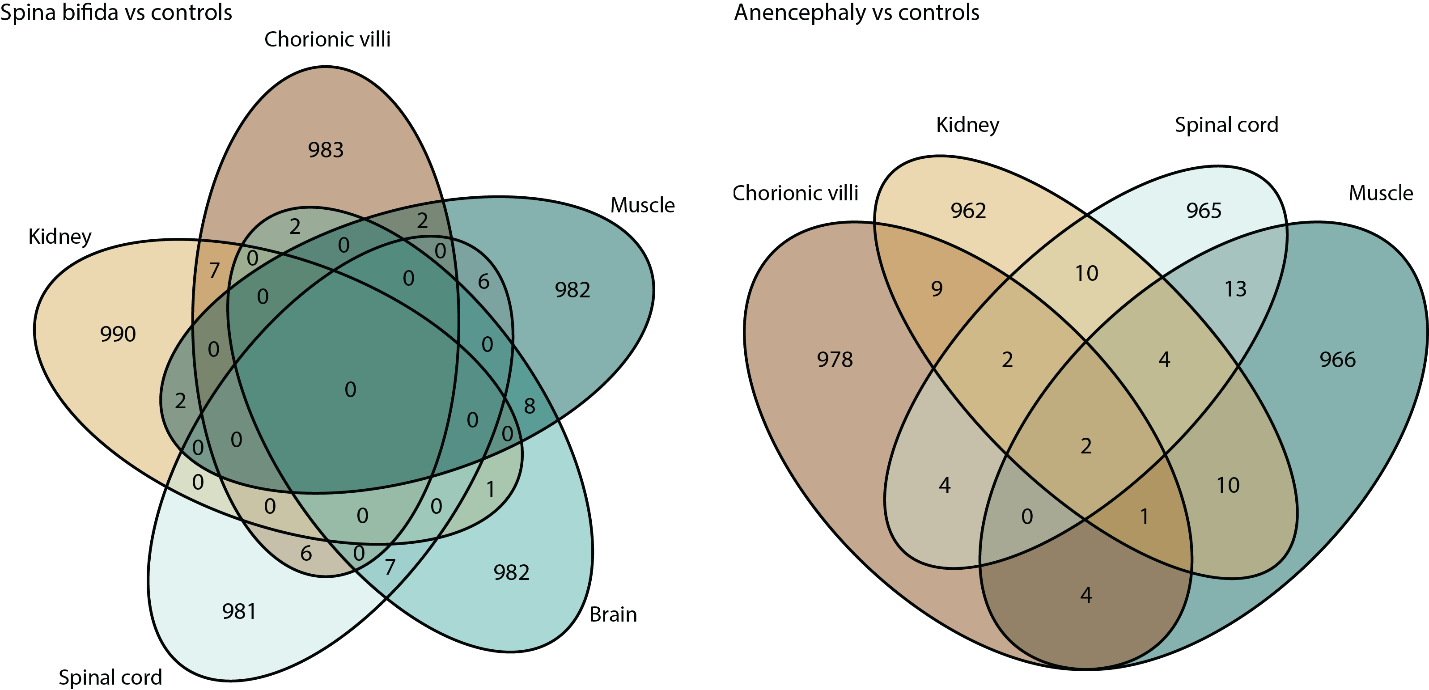


**Fig. S6 Cases used in the gestational age matched comparison.** A subgroup of 19 mixed control and anencephaly cases were used in a supplementary gestational age-matched comparison of non-spina bifida to spina bifida samples. *P*-value calculated by Mann-Whitney test.

**
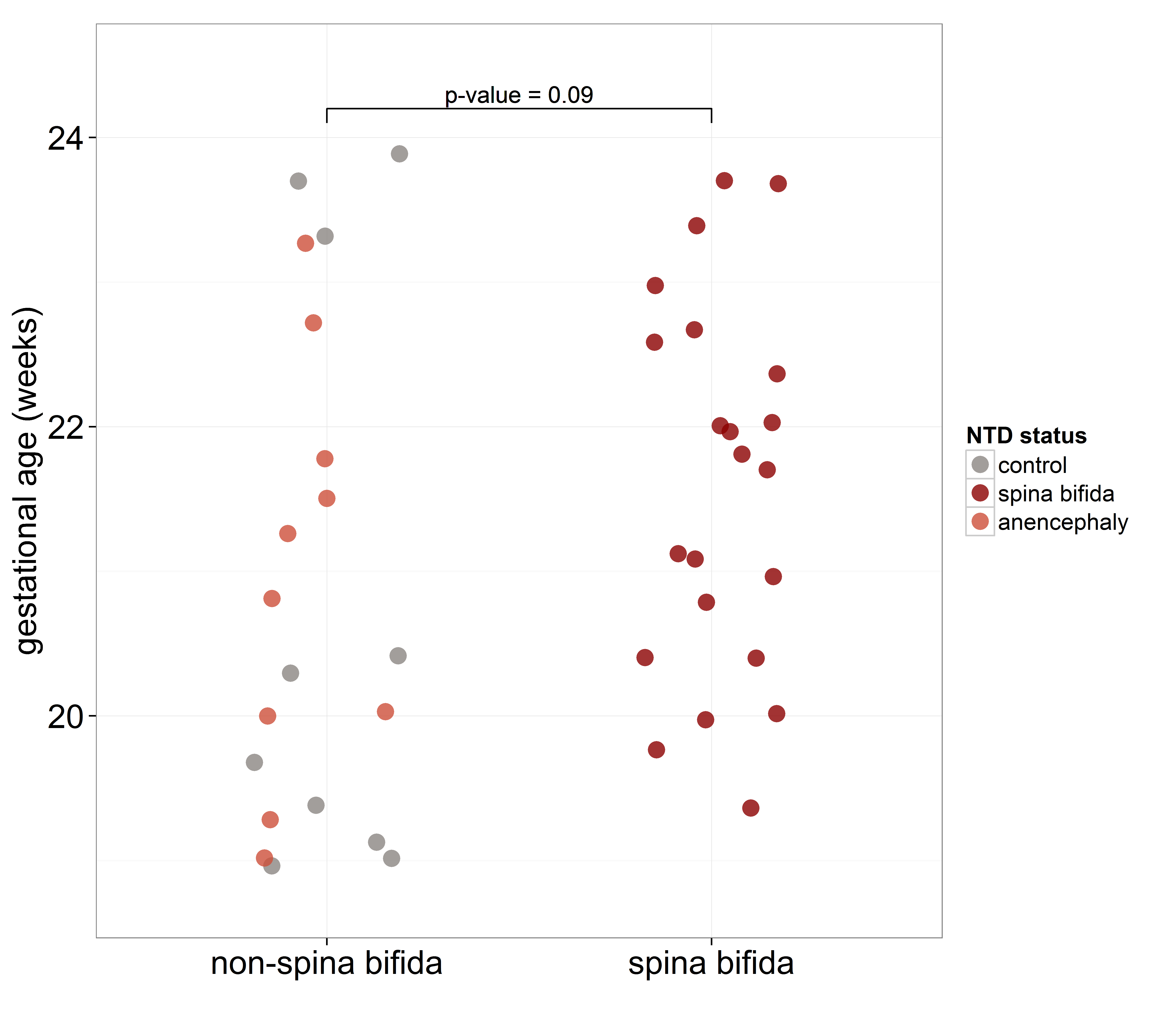
**

**Fig.S7 Pyrosequencing of cg1098862 in chorionic villi.** Pyrosequencing was performed to follow up differential methylation (DM) at cg1098862, identified in the 450k array comparison of anencephaly to controls in chorionic villi. This CpG is located 146 bps upstream of poly (ADP-Ribose) Polymerase 1 (*PARP1)*, a gene shown to regulate trophoblast differentiation, involved in ADP-ribosylation of histones and loss of function is associated with invasive and metastatic properties in cancer. The differential DNAm was validated in the set of samples run on the 450k array (*p*=0.000006) and replicated in the smaller, extended set of samples (*p*=0.02). % DNA methylation plotted was adjusted for fetal sex and gestational age.


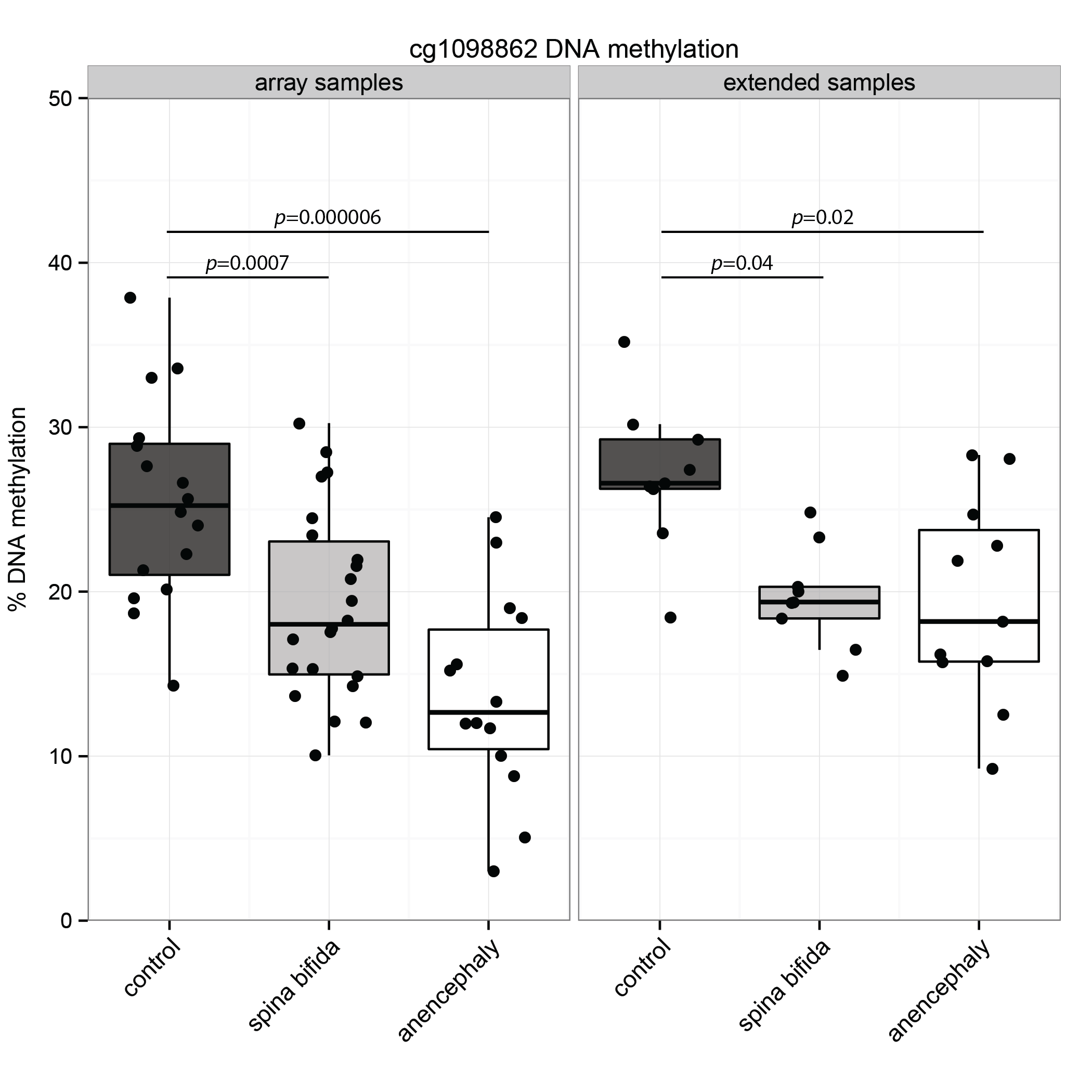


**Fig.S8 Pyrosequencing of cg02413938 in chorionic villi.** Pyrosequencing was performed to follow up differential methylation (DM) at cg02413938, identified in the 450k array comparison of anencephaly to controls in chorionic villi. This CpG is located 21 bps upstream of ectoplasmic specialization protein like (*ESPNL*), a gene with a role in proliferation and invasion in melanoma. An A/G SNP (rs64315796) 6 bps downstream of the target CpG site was included in the pyrosequencing assay. (A) Differential DNAm was validated in the set of samples run on the 450k array (*p*=0.05), but not replicated in a smaller, extended set of samples. (B) We identified significant differential DNAm at cg02413938 by genotype of rs64315796, including both array and extended samples. There was unequal distribution of genotypes by NTD status in the array cohort, which likely accounts for the DM picked in the 450k array analysis. % DNA methylation plotted was adjusted for fetal sex and gestational age.


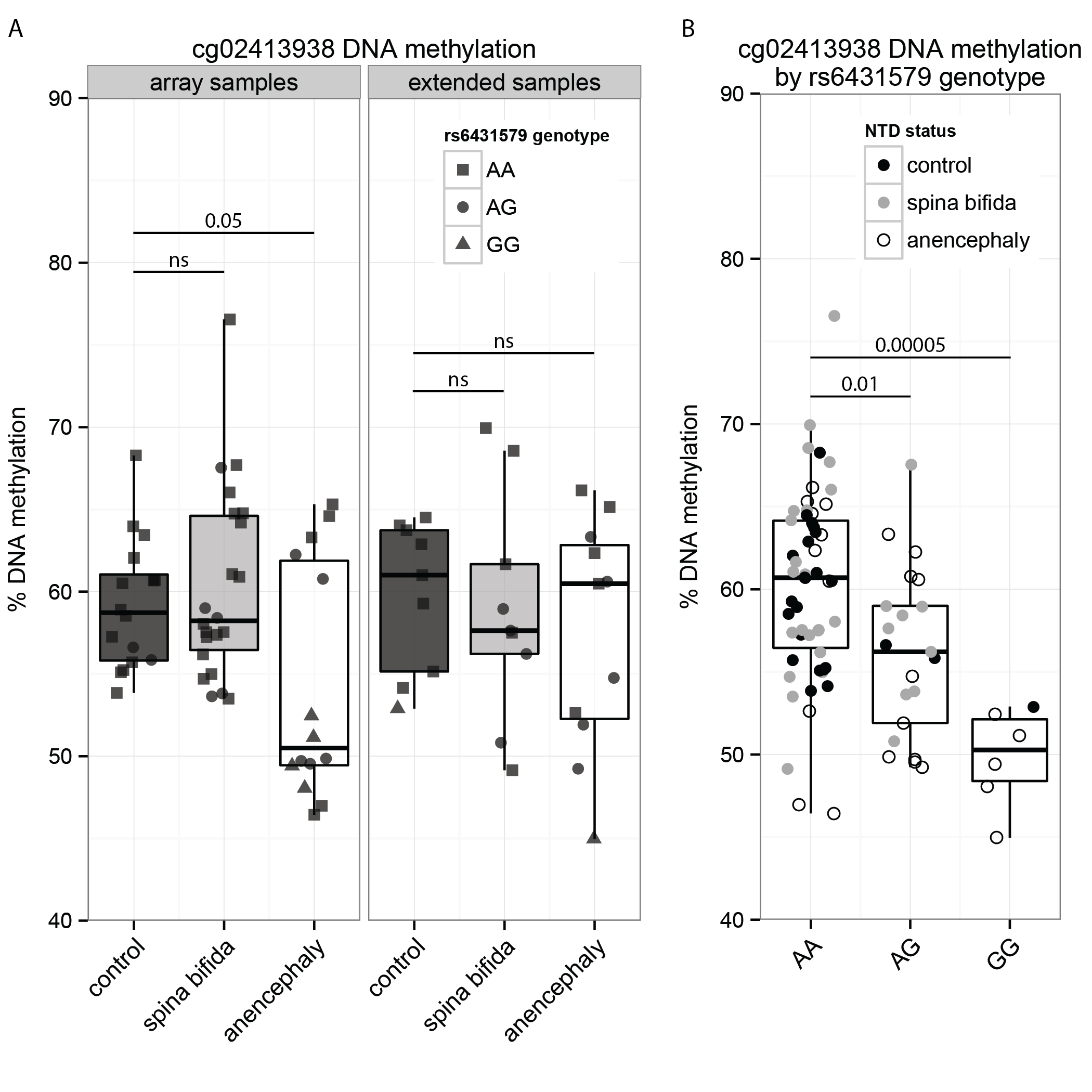

Supplement: Supplementary file 2 — 10.1186/s13072-016-0054-8 Fig. S1 is a box plot of array-wide average DNAm per sample. Fig. S2 is a box plot of the percentage of outlier CpG sites per sample. Fig. S3 are volcano plots of DM between anencephaly and controls at cleaned 450k CpG sites. Fig. S4 is a Venn diagram overlapping the top 1000 DM CpG sites by NTD status for each tissue. Fig. S5 is a Venn diagram overlapping the top 1000 DM CpG sites by tissue for each NTD status. Fig. S6 is a plot of the cases used in the gestational age-matched non-SB vs. SB comparison. Fig. S7 is a box plot of DNAm in chorionic villi measured by pyrosequencing in the follow-up of cg1098862. Fig. S8 is a box plot of DNAm in chorionic villi measured by pyrosequencing in the follow-up of cg02413938. [file 13072_2016_54_MOESM2_ESM.docx]
